# The Need to Address Fragmentation and Silos in Mortality Information Systems: The Case of Ghana and Peru

**DOI:** 10.3389/ijph.2022.1604721

**Published:** 2022-12-14

**Authors:** Daniel Cobos Muñoz, Carmen Sant Fruchtman, Janet Miki, Javier Vargas-Herrera, Sarah Woode, Fidelia A. A. Dake, Benjamin Clapham, Don De Savigny, Emmanuel Botchway

**Affiliations:** ^1^ Swiss Tropical and Public Health Institute (Swiss TPH), Basel, Switzerland; ^2^ Epidemiology and Public Health Department, University of Basel, Basel, Switzerland; ^3^ Vital Strategies, New York, NY, United States; ^4^ Departamento de Medicina Preventiva, National University of San Marcos, Lima, Peru; ^5^ Ghana Statistical Service, Accra, Ghana; ^6^ Regional Institute for Population Studies, University of Ghana, Accra, Ghana; ^7^ Birth and Death Registry, Accra, Ghana

**Keywords:** cause of death, mortality surveillance, civil registration and vital statistics, mortality statistics, process mapping, social network analysis

## Abstract

**Objectives:** We aimed to understand the information architecture and degree of integration of mortality surveillance systems in Ghana and Peru.

**Methods:** We conducted a cross-sectional study using a combination of document review and unstructured interviews to describe and analyse the sub-systems collecting mortality data.

**Results:** We identified 18 and 16 information subsystems with independent databases capturing death events in Peru and Ghana respectively. The mortality information architecture was highly fragmented with a multiplicity of unconnected data silos and with formal and informal data collection systems.

**Conclusion:** Reliable and timely information about who dies where and from what underlying cause is essential to reporting progress on Sustainable Development Goals, ensuring policies are responding to population health dynamics, and understanding the impact of threats and events like the COVID-19 pandemic. Integrating systems hosted in different parts of government remains a challenge for countries and limits the ability of statistics systems to produce accurate and timely information. Our study exposes multiple opportunities to improve the design of mortality surveillance systems by integrating existing subsystems currently operating in silos.

## Introduction

Since the start of the COVID-19 pandemic in the year 2020, substantial attention has been put on the count of deaths to understand how the pandemic is evolving [1]. Many countries are struggling to produce reliable estimates of the number of deaths and their causes at a pace that can inform policy decisions. This is partly because their mortality information is segregated across health, civil registration, and other sectors, and data are incomplete, and frequently classified as being of low quality [2, 3].

The majority of low and middle-income countries lack a comprehensive birth and deaths registration system, with only 45 percent of global deaths registered in official registers [4]. Improvement over the years has been slow [5–7], partly because of the difficulty of working across administrative silos in government. Deaths and their details can be recorded in multiple formal and informal information subsystems, such as national identity agencies, social security services, civil registration, health services, health and demographic surveillance sites, mortuaries, coronial and forensic services, burial administrative systems, police, insurance systems, funeral homes, or cemetery records. These subsystems are sitting in multiple ministries across the government or private sector with traditional administrative boundaries forcing them to operate in silos. Some of the informal mortality surveillance sub-systems, such as funeral homes, are not regulated or monitored in some countries and the data collected by then is not captured by formal mortality surveillance systems. As a result of this fragmentation, none of these subsystems has a full picture of the complete mortality statistics of the population in the country since they capture information from different and partially overlapping population groups. Subsystems concerned with legal identity, civil registration, or health data, for example, are supposed to be nested within civil registration and vital statistics information systems [8]. However, these systems are often disconnected and not integrated, which leads to underperforming information flows and inefficiencies in data management [9, 10].

The need for accurate and timely data to inform the response to the COVID-19 pandemic has driven countries to develop short-term solutions to capture overall and cause-specific mortality [11]. It has also exposed data systems that could serve as opportunities to estimate excess mortality and understand its trends over time. As a first step towards integrating mortality data sources to build comprehensive mortality surveillance systems, cataloguing all potential information sources and understanding how they interact or overlap with each other will be essential. To our knowledge, this is one of the first studies to explore the potential of integrating these sources of recorded death information into the wider CRVS system. In this paper, we aim to understand the information architecture of the mortality surveillance system and its degree of integration in two diverse lower middle-income countries, Ghana and Peru, before the start of the COVID-19 pandemic. We use an enterprise architecture and systems thinking approach to understand how these systems operate, what their information requirements are, and the technology used to host their information [12, 13]. Preliminary results of this study were describe elsewhere in the form of a working paper [14].

## Methods

We conducted a cross-sectional study designed to map and catalogue information sub-systems collecting mortality data in Ghana and Peru. Subsystems were defined as institutions or information systems that were recording any information about a death event, including the cause of death. Subsystems were identified and described using enterprise architecture process maps [15]. In these maps, the sequence of activities required in the process to record the fact or the cause of death in each of the systems is described alongside the stakeholders implementing them. They also captured the different documents (forms, and records) used in each system to record information about the death event, as well as a list of the data elements (e.g., name, age, sex, cause of death) captured in the different documents. The landscape of mortality information subsystems and their business and information architecture was documented through a systematic document review and unstructured interviews between September 2018 and January 2019.

### Study Settings

Peru and Ghana represent different states of CRVS system maturity in two very different contexts (Table 1).

**TABLE 1:** General characteristics of the civil registration and vital statistics systems in Peru and Ghana. The need to address fragmentation and silos in mortality information systems: The case of Ghana and Peru, 2019.

Indicator	Peru	Ghana
Total Population in the country (2017)	31.2 million	28.9 million
Birth registration completeness	95%^a^	65%
Death registration completeness	70%^a^	23%
Level of decentralization of the CRVS system	Decentralized	Decentralized
Leading CRVS institution	Civil registration authority (RENIEC)	Births and Deaths Registry
Most recent year with published vital statistics	2021 [51]	N/A
National Identity coverage (%)	99.3%^a^	N/A

^a^ https://www.inei.gob.pe/media/principales_indicadores/libro_bol_esp_24_4.pdf and https://www.inei.gob.pe/estadisticas/indice-tematico/poblacion-y-vivienda/

Peru has a long history of CRVS improvements, resulting in a functional and strong CRVS system [16] with a high level of decentralization. The civil registration agency (RENIEC from the acronym in Spanish) or the municipal offices operate local civil registration offices. At the time of the study, there were 54 registry offices, 171 auxiliary registry offices, and 4,881 municipal registries. The majority of the offices register events and issue certificates using an electronic system named SIRCM. The system also has auxiliary registry offices in hospitals and health centres to bring registration services closer to users of the health sector. Municipal registry offices are local government offices that register vital events and issue certified copies of death registrations [17]. Death events are reported in the health system through either “SINADEF” (electronic system)or using the paper version of the MCCD (60% of deaths using the paper system) [18, 19].

Still considered a low performing CRVS system, Ghana is currently conducting a large-scale reform of their CRVS system as a result of a comprehensive assessment conducted in 2014 [20, 21]. Ghana ’s 10 regions are divided into a total of 216 districts with 426 functioning Birth and Death Registration (BDR) offices across the country.

The share of births and deaths captured by the CRVS system in Ghana is low [19, 22] despite the fact that the Registration of Births and Deaths Act 301 (1965) dictates that all births, deaths, and foetal deaths must be registered. There is a low coverage of vital events registration owing to a high degree of complexity and bureaucracy in the CRVS operations, insufficient awareness among the public on the importance of having timely and reliable vital statistics, limited geographical access for rural communities, limited funding, scarce human resources, and direct and indirect registration cost for the users of the system [19].

### Data Collection

The researchers in each country identified relevant subsystems for mortality information systems at the national and local levels within their routine activities in the mortality information system. A snowball sampling approach was used to identify further unofficial information systems. The lead researchers in each country arranged face-to-face visits to each identified institution to conduct unstructured interviews and collect relevant documents. Twenty-two interviews were conducted in total, of these nine took place in Ghana and 13 in Peru. The document review included 22 documents and forms from Ghana and 42 from Peru. In Ghana, EB, a male director of the Births and Deaths Registry and SW a female statistician within Ghana Statistical Service led the data collection. CSF a female researcher and DCM a male researcher, both from the Swiss Tropical and Public Health Institute, supported data collection activities in Ghana. In Peru, JM a female project manager for Vital Strategies working within the mortality reforms and JV a male researcher at the Universidad San Marcos, and also involved in the mortality surveillance reforms, led the data collection design and JM conducted all interviews and workshops.

Face to face unstructured interviews with personnel from different institutions were conducted. Researchers’ interview notes were typed and organised into summaries after each interview. Detailed information about the institutions that were visited for unstructured interviews and document review is compiled in Table 2.

**TABLE 2:** Overview of the institutions and actors included in the data collection activities in Ghana and Peru. The need to address fragmentation and silos in mortality information systems: The case of Ghana and Peru, 2019.

Visited organisation	Location
City Council	Accra, Ghana
RIDGE Hospital	Accra, Ghana
Accident and Emergency Centre (Public hospital)	Accra, Ghana
Korle Bu mortuary	Accra, Ghana
Imam (religious leader)	Accra, Ghana
Orthodox church	Accra, Ghana
Private cemetery	Accra, Ghana
Public cemetery	Accra, Ghana
Private funeral home	Accra, Ghana
MINSA Hospital	Lima, Peru
ESSALUD Hospital	Lima, Peru
Hospital de las Fuerzas Armadas y Policiales	Lima, Peru
Private Clinic	Lima, Peru
RENIEC	Lima, Peru
Cemetery	Lima, Peru
OGTI-MINSA	Lima, Peru
INEI	Lima, Peru
Centro de Salud San Pablo de Tushmo	Ucayali, Peru
Municipalidad de centro poblado de San Jose Yarinacocha	Ucayali, Peru
Rural municipality—burial site	Ucayali, Peru

The forms and registers used to record the deceased information were identified and blank copies of the registration forms were collected, if available. The nature of the documentation was recorded in a pre-defined data collection tool in Excel®. Briefly, we captured general information about the sub-systems, the data elements collected in each form, as well as the sender and receiver of each piece of information. Information subsystems were catalogued into producers or consumers of vital statistics information depending on their primary role in the system. We also reviewed the specific data elements that each of these forms were capturing and these were categorized as essential or non-essential. Essential mortality data elements were considered to be the following 12 elements: Full name, Sex, Marital Status, Date of birth, Date of death, Age at death, Cause of Death (COD), Name of medical practitioner certifying COD, Date of COD, Date of registration, Registration District and Registrar name.

### Data Analysis

Researchers’ written interview notes were typed and organised into summaries of each interview and workshop. We synthesized the information using several systems thinking tools and approaches. We developed process maps [23] in Bizagi Modeler [24] and used standard Business Process Mapping Notation 2.0 [25] to visualize the flow of information across subsystems and the sequence of activities implemented by each institution. One country-specific map illustrated all subsystems and their interrelationships.

The flow of information among the various subsystems were visualized in an adapted Social Network Analysis diagram [26]; specifically, a “Force-directed graph” [27] visualization in Power BI was used. In this diagram, nodes (circles) represent the information subsystems and the arrows shows the flow of information between them, showing the direction of the exchange. The two visualizations, the country-specific process map and the force-directed graph, analysed the degree of integration and interconnections in the network of sub-systems as well as the flow of information through them.

## Results

In both Ghana and Peru, several information subsystems were identified in which the details of deaths were recorded and not shared with other systems. In these fragmented information constellations, families were the only common connection between several of these information systems, leading to an administrative burden for the families.

### Peru

Of the eighteen information subsystems identified in Peru (Table 3). Nine of them were functioning at the local level and collecting data for each individual vital event. The other nine were national institutions primarily transmitting and storing aggregate information.

**TABLE 3:** Characteristics of the different entities identified as part of the mortality information sub-systems in Peru (Adapted from Cobos et al. [14]. The need to address fragmentation and silos in mortality information systems: The case of Ghana and Peru, 2019.

System type	Name	Level	Role in the system^a^
Health services	ESSALUD facility	Local	Producer
	Military Force facility	Local	Producer
	Private clinic	Local	Producer
	Public facility	Local	Producer
	Ministry of Health (MINSA)	National	Consumer
Government administration	Morgue	Local	Producer
	RENIEC local office	Local	Producer
	Municipality	Local	Consumer
	Burial system	Local	Producer
	National Statistics Office (INEI)	National	Consumer
	Justice system	National	Consumer
	Military force central office	National	Producer
	Comprehensive Health Insurance (SIS)	National	Consumer
	Peruvian Government	National	Consumer
	National Civil Registration Authority (RENIEC)	National	Consumer
	National social security system (ESSALUD)	National	Consumer
Family		Local	Producer and consumer
Funeral homes and Cemeteries		Local	Producer

^a^ Roles in the subsystem refer to the primary function of the institution in the system. Producers are actors participating in the collection and transmission of information about the vital event and/or the generation and dissemination of vital statistics. Consumers are those actors whose primary interest is the use of either individual level data or aggregated statistics about vital events.

**FIGURE 1:**
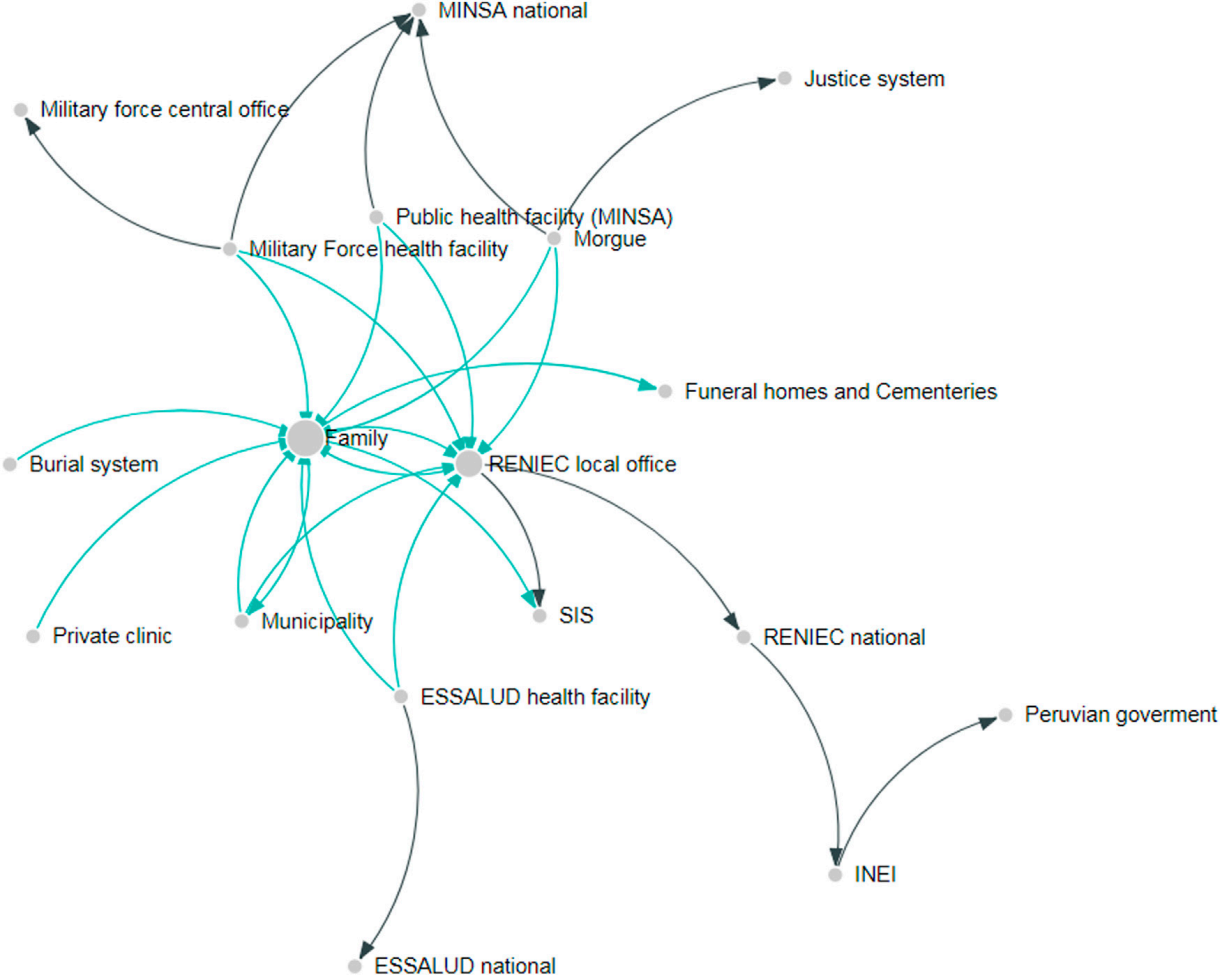
Information architecture in Peru. Each node represents an institution involved in recording or transmitting information about death events. The size of the node represents the number of incoming interactions in the network. The arrows represent the direction of the information flow. Green arrows represent interaction at the local level, while black arrows represent interaction among national institutions. The need to address fragmentation and silos in mortality information systems: The case of Ghana and Peru, 2019.

Figure 1 shows the design of the mortality surveillance system in Peru. Several subsystem nodes are located on the edge of the network, receiving information but not sharing this with any other system, which exposes a substantial degree of fragmentation in the overall mortality inforamtion ecosystem. These nodes represent the national headquarters of the leading institutions in each of the sectors involved in generating vital statistics (e.g., health, police or civil registration authority) and there are no links showing information sharing among them.

Information exchange and interactions among subsystems were greater at the local level. Two main nodes integrating information in the system were identified: the family and the local civil registration office (RENIEC office).

The family of the deceased has the highest number of incoming and outgoing information links in Peru. Even though there exist information sharing among health facilities and registration offices, the process maps describing the sequence of activities in the system show that the family are demanded to complete the different administrative tasks in person by visiting the multiple offices (see Figure 1). The local RENIEC office also functions as a major integration hub, receiving information from public and private health facilities using SINADEF for medically certified deaths and pushing the information to some of the other systems.

In terms of administrative documentation, a total of 42 documents recording information about the death event were identified across institutions. 36 documents recorded information on individual death events and six transmitted or stored aggregated figures. Most of them were exclusively paper forms including different versions being used different institutions.

Of the essential and non-essential data elements, none of the 42 forms collected all 12 essential elements. The necropsy report from the morgue was the document with the highest number of essential and non-essential data elements captured (*n* = 32).

### Ghana

**FIGURE 2:**
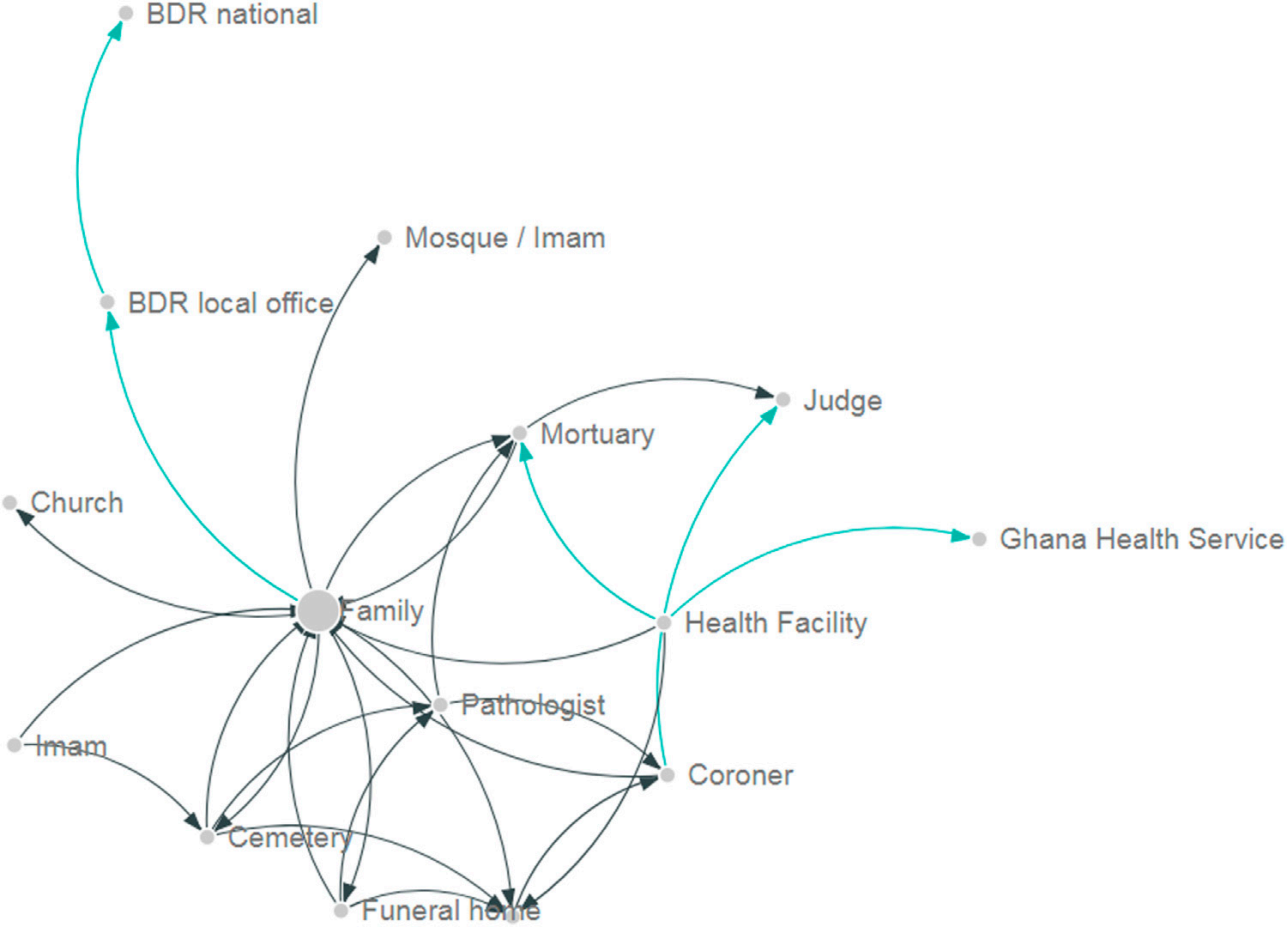
Information architecture in Ghana. Each node represents an institution involved in recording or transmitting information about death events. The size of the node represents the number of incoming interactions in the network. The arrows represent the direction of the information flow. Green arrows represent interaction at the local level, while black arrows represent interaction among national institutions. The need to address fragmentation and silos in mortality information systems: The case of Ghana and Peru, 2019.

In Ghana, 16 information subsystems were identified (Table 4), being 13 of them local subsystems recording data of each death event and three national systems primarily collecting aggregate information about deaths in the country. When visualizing the information architecture of the system (Figure 2), the family, which is represented in the central node, acts as the main hub of the information ecosystem around mortality in Ghana. In order the achieve the registration of the death event, the family would need to interact with health facilities, funeral homes, police in some instances, BDR offices and local government officials. In the worst-case scenario, families would have to interact with up to eight different systems before they can complete all administrative and other procedures.

**TABLE 4:** Characteristics of the different entities identified as part of the mortality information sub-systems in Ghana Peru (Adapted from Cobos et al. [14]. The need to address fragmentation and silos in mortality information systems: The case of Ghana and Peru, 2019.

System type	Name	Level	Role in the system^a^
Health services	Health Facility	Local	Producer
	Coroner	Local	Producer
	Mortuary	Local	Producer
	Pathologist	Local	Producer
	Ghana Health Services (GHS)	National	Consumer
Government administration	Police	Local	Producer
	Birth and Death Registration (BDR) local office	Local	Producer
	Judiciary system	National	Consumer
	BDR national	National	Consumer
Funeral homes and Cemeteries	Funeral home	Local	Producer
	Mosque, Imam, Church	Local	Producer
Family	Family	Local	Consumer

^a^ Roles in the subsystem refer to the primary function of the institution in the system. Producers are actors participating in the collection and transmission of information about the vital event and/or the generation and dissemination of vital statistics. Consumers are those actors whose primary interest is the use of either individual level data or aggregated statistics about vital events.

The BDR national database only captured those deaths in which the family physically attended a BDR office to formally complete the registration process. As a consequence, deaths occurred outside health facilities which typically are not medically certified by a trained physician, health facilities deaths for which the family did not pursue registration or death recorded in the judiciary or police system (deaths due to intentional or unintentional injuries) will not find their way into the BDR database. On the other side, a death event could be recorded in multiple systems leading to duplication and inefficiencies.

22 forms and registers were identified as recording information about the death event. Most documents (*n* = 20) were designed to capture information for each death event including some of them information about the cause of death. 17 of these documents were paper forms, four were in a digital system, and one was supported by both systems. As occurred in Peru’s administrative data, most forms did not include all essential elements required to register a death. The registration book from BDR was the document that had the most essential elements (*n* = 11), and only lacked the date of birth.

## Discussion

Our study shows that the mortality information systems in Peru and Ghana are similarly highly fragmented across different institutional silos and they are strongly dependent on families to register the death event. The implications are considerable as this contributes to a lack of completeness and inequities in death registration. Wealthier citizens from urban areas with access to the health system will be more likely to register deaths, leading to a misrepresentation of people’s needs in vital statistics, as has been seen with birth registrations [28].

The country systems portrayed in this analysis—one being a relatively well-performing system and the other a less mature CRVS system—were highly fragmented with a multiplicity of unconnected data silos. Mortality information silos represented the parallel operations of the different subsystems involved in the wider mortality surveillance ecosystem. At the same time, having multiple actors collecting information about deaths and causes of death can be seen as an opportunity if integrated with the mainstream system. Religious institutions, cemeteries, or funeral homes could play a significant role in capturing information about death events in countries where neither the civil registration nor the health sector has a complete record of all deaths. They represent untapped opportunities for integration towards more complete mortality surveillance systems. Examples of the integration of non-traditional sources of mortality statistics have been reported for HIV or during crises [29–31].

Despite the global consensus on the importance of vital registration, the completeness of CRVS systems is a concern for most LMICs [28, 32–34], especially for death registration. Information about causes of deaths, when available, is of low quality in many settings and limits its use for public health policymaking [35]. This has been demonstrated during the COVID-19 pandemic. Timely and complete mortality data for COVID-19 is vital for countries to plan and implement COVID-19 control strategies and plans and to understand the impact it is having on different populations [36], yet a lack of such data has affected countries’ ability to contain and control the pandemic [37]. Addressing this gap is essential for the social and economic development of every country [38, 39].

Two of the fundamental root causes of the low vital event registration completeness in CRVS systems of LMICs are inefficient CRVS operations and systems passively waiting for the family to complete the different administrative processes to register vital events [35, 40]. The lack of integration of these systems and their passivity, where the system relies on families to physically visit a location several times to register a death, has likely hampered efforts to improve CRVS as a whole. Even in well-performing systems like the one in Peru, where the two main subsystems (health and civil registration) are digital and already sharing some information, there is a need for the family to move to the relevant offices to complete the administrative process [41, 42]. This leads to bottlenecks in improving the system, as the responsibility for registering deaths partially remains on the individuals and families, who may not be able to complete the process. For instance, during COVID-19, an inability for people to travel to offices, as well as factors like shorter office hours or disruptions in rural outreach for registrations, were challenges in multiple countries [43].

The lack of integration of the systems in Peru and Ghana could lead to inefficiencies and could enable double-counting, undercounting, or other inaccuracies and inconsistencies. Based on our results, the details of one death would need to be recorded up to seven times in different systems capturing different data elements. As a consequence, none of the systems would have the complete information about the death event and would be impossible to identify discrepancies. This fragmentation and incompleteness have also been reported for road traffic deaths in Uganda [44], HIV patients in South Africa [45] and as a major challenge for data quality in health information systems in LMICs [46]. Modern database technology offers a clear opportunity to integrate these different information systems in Ghana and Peru, given in the current digitization efforts in both countries. Nevertheless, technology alone, will not solve these challenges. Reforms will be required to understand the root causes of the inefficiencies to redesign the systems and create more integrated and complete systems.

The integration of different information systems within CRVS systems will not only be a technical challenge but a systems one [47]. However, the integration alone will not solve the challenge of increasing the completeness of mortality statistics. We believe it will be essential for countries to combine interventions and system redesign. In a previous study, we identified several ways to mitigate this weakness [40]. By redesigning death registration to be proactive, decentralized, and less bureaucratic, it was possible to increase death registration coverage in Ghana and Tanzania. Acknowledging the systemic challenges to integration will be of great importance if countries incorporate additional information sources, like verbal autopsy, into the routine CRVS. Verbal autopsy is the main resource low-income countries are using to bridge the data gaps in registration of deaths and cause of death in communities, especially in rural settings [48, 49]. This will be a great opportunity but also a challenge, as it will increase the complexity of the information systems. In countries choosing to implement verbal autopsy to serve CRVS purposes, the integration of information will be essential.

This study also emphasizes the challenges of developing omnibus CRVS implementation software [50]. In Peru and Ghana, the existence of a large variety of forms and different data elements that were collected across the subsystems would make it challenging to develop national standard processes. Peru and Ghana are now in the process of redesigning their CRVS systems, and as they do so, this study has shown they will need to reconsider the architecture of their information systems. Local efforts to develop IT applications and tools could help producers and consumers to rethink the rationale behind the data that they currently collecting and the use of the best mortality data sources. This could allow them to leverage existing resources and increase the quality and coverage of their systems.

### Conclusion

As countries struggle to collect timely and reliable mortality statistics to combat COVID-19, mortality surveillance systems are proving to be a great ally, as well as a pending task in many countries. With this study, we aimed to bring some clarity into the mortality information gap by mapping all the subsystems recording deaths. The study makes the case for further research in other contexts to piece together a path towards integrated and efficient mortality surveillance systems.

The study showed that none of the multiple sub-systems collecting information about death events in Peru or Ghana could provide a complete picture of mortality statistics nationwide or for the different population groups in the country. The different sub-systems are recording death events in partially overlapping population groups and our analysis reveals that integration across the various information sub-systems would increase death registration completeness.

Globally, death registration is severely lacking [4]. The use of systems thinking tools, such as process mapping, should be employed as an initial step to elucidating the fragmentation and opportunities for harmonization across mortality registration systems and various stakeholders, as has been demonstrated in this study. More integrated, efficient, and complete mortality registration systems are needed in many countries and contexts, but in doing so, digitalization needs to be carefully designed in order to avoid replicating inefficient paper systems in digital format.

## Data Availability

All the data used in this study are available from the authors upon reasonable request.

## Abbreviations

BDR, Births and Deaths Registry; COD, Cause of death; RENIEC, Registro Nacional de Identificación y Estado Civil; CRVS, Civil registration and vital statistics; LMIC, Low and middle-income countries; SINADEF, Sistema Informático Nacional de Defunciones; BDR, Birth and Death Registry; INEI, Instituto Nacional de Estadística e Informática; SIS, Seguro Integral de Salud; MINSA, Ministerio de Salud.
